# Großer Zeh – kleines Übel? Wenn eine ambulante Operation zu einem stationären Aufenthalt führt

**DOI:** 10.1007/s00104-025-02250-x

**Published:** 2025-02-13

**Authors:** A. F. Erdogan, A. Oberhuber

**Affiliations:** Klinik für Vaskuläre und Endovaskuläre Chirurgie, Universitätsklinikum Münster (UKM), Albert-Schweitzer-Campus 1, Waldeyerstraße 30, Geb. W 30, 48149 Münster, Deutschland

## Anamnese

Ein 89-jähriger Patient stellte sich notfallmäßig in unserer Ambulanz mit zunehmenden Ruheschmerzen im linken Bein und Fuß sowie Schmerzen am Digitus I des linken Fußes vor. Zwei Tage zuvor war aufgrund eines Unguis incarnatus am Digitus I des linken Fußes eine ambulante Emmert-Plastik unter Anwendung pneumatischer Kompression zur Blutleere durchgeführt worden.

Bei der Vorstellung berichtete der Patient über starke Wundschmerzen im Bereich der Operationswunde, die bereits am Abend des ambulanten Operationstages aufgetreten waren. Am Folgetag verschlimmerten sich die Schmerzen im linken Fuß und führten zu persistierenden nächtlichen Ruheschmerzen, die das gesamte linke Bein und den Fuß betrafen. Unter den Vorerkrankungen ließen sich eine koronare Zweigefäßerkrankung (2-Gefäß-KHK), eine hypertensive Herzerkrankung, eine chronische Nierenerkrankung im Stadium 3 (KDIGO) sowie eine Kolonteilresektion bei Kolonadenomen feststellen. Bereits präoperativ klagte der Patient über eine verkürzte schmerzfreie Gehstrecke infolge von Wadenklaudikation.

## Klinischer Befund

**Abb. 1:**
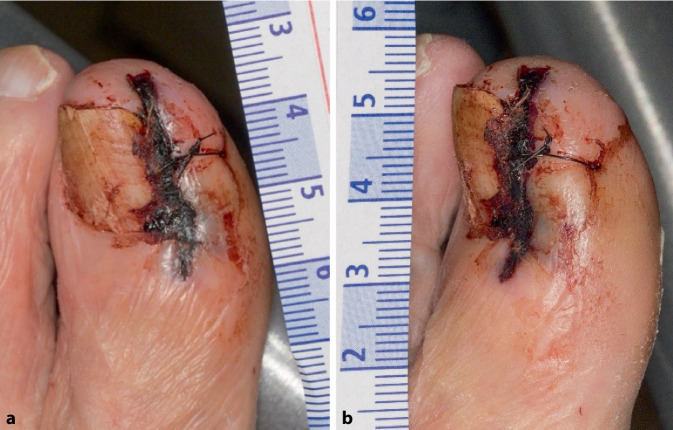
Digitus I des linken Fußes präoperativ, **a** Ansicht von ventral, **b** Ansicht von medial

In der klinischen Untersuchung zeigte sich ein stark schmerzgeplagter Patient in stabilem Allgemeinzustand. Beide Beine und Füße präsentierten sich warm, ohne erkennbare Temperaturunterschiede oder Umfangszunahmen, mit einer Rekapillarisierungszeit von < 2 s. Jedoch zeigte sich der Digitus I am linken Fuß gerötet und leicht überwärmt. Die Operationswunde war nekrotisch (Abb. 1).

## Diagnostik

Es ließ sich lediglich ein abgeschwächter Leistenpuls beidseits bei erloschenen peripheren Fußpulsen palpieren. Ein sensomotorisches Defizit blieb aus. Die Durchführung eines *Knöchel-Arm-Index* (Ankle-brachial Index, ABI) wurde aufgrund starker Schmerzen nicht von dem Patienten toleriert. In der Duplexsonographie des linken Beckens und Beins zeigte sich eine hochgradige exzentrische Stenose der A. femoralis communis (AFC). Sowohl die A. femoralis superficialis (AFS) als auch die A. poplitea (APOP) wiesen starke atherosklerotische Veränderungen mit mindestens einer hochgradigen Stenose auf. Die A. tibialis posterior (ATP) zeigte nur ein sehr abgeschwächtes, monophasisches Flusssignal, während in der A. tibialis anterior (ATA) kein Fluss darstellbar war.

## Wie lautet Ihre Diagnose?

## Verlauf

Es erfolgte die Indikationsstellung zur Thrombendarteriektomie (TEA) der Femoralisbifurkation links sowie einer Angiographie in Interventionsbereitschaft. Bei der Endarteriektomie der Femoralisgabel unter Einbeziehung der distalen A. iliaca externa zeigte sich die vorab duplexsonographisch dargestellte Stenose als funktioneller Verschluss. In der Angiographie nach distal zeigte sich eine hochgradige Stenose der A. poplitea, welche mit einer Lithoplastie behandelt wurde. Am Unterschenkel fand sich eine Eingefäßversorgung durch die A. fibularis, welche die A. tibialis posterior über ein Kollateralisierungsnetz auffüllte.

Postoperativ äußerte der Patient eine sofortige Besserung der Bein- und Fußschmerzen bei einem tastbaren Puls über der linken A. poplitea. Die postoperative ABI-Messung ergab einen Index von 0,9 über der linken ATP. Eine perioperativ begleitende intravenöse Alprostadil-Therapie wurde bis zum 6. postoperativen Tag weitergeführt. Somit konnte der Patient bei einem komplikationslosen postoperativen Verlauf mit einer erkennbaren Heilungstendenz aller Operationswunden entlassen werden.

**Diagnose:** Periphere arterielle Verschlusskrankheit (pAVK) des Ober- und Unterschenkeltyps links, Stadium IV nach Fontaine

**Abb. 2:**
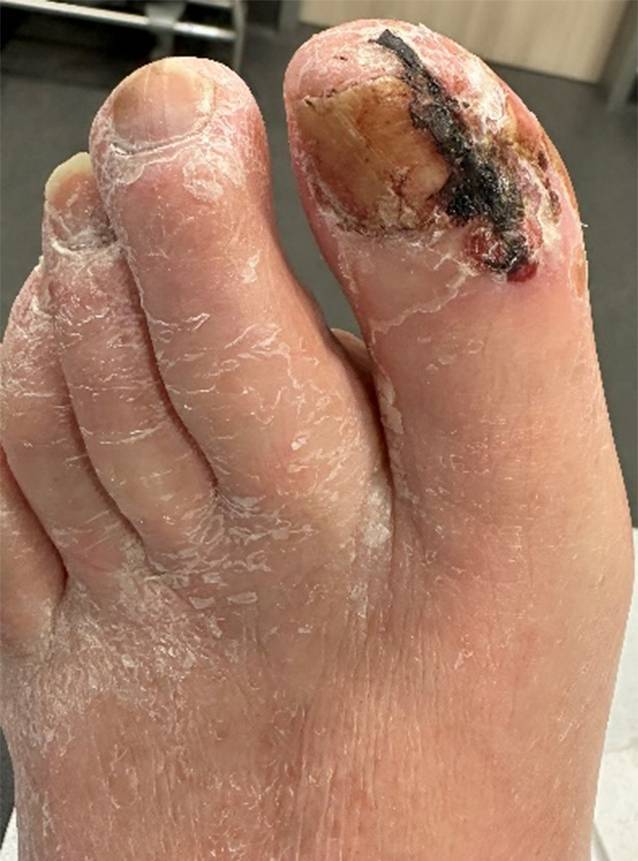
Digitus I des linken Fußes, 17 Tage nach Revaskularisation, Ansicht von ventral

Sechs Wochen später sahen wir den Patienten zur Verlaufskontrolle in unserer Ambulanz. Ruheschmerzen und Klaudikation im linken Bein wurden verneint. Der postoperative Pulsstatus blieb bei erhaltenem Leistenpuls beidseits und Poplitealpuls links unverändert. Die Operationswunde der Emmert-Plastik war fast komplett abgeheilt (Abb. 2 und 3). Duplexsonographisch konnte ein triphasisches Flusssignal in der linken AFC und AFS sowie ein biphasisches Signal in der APF erfasst werden. Im P‑III-Segment der A. poplitea zeigte sich ein monophasisches Signal.

**Abb. 3:**
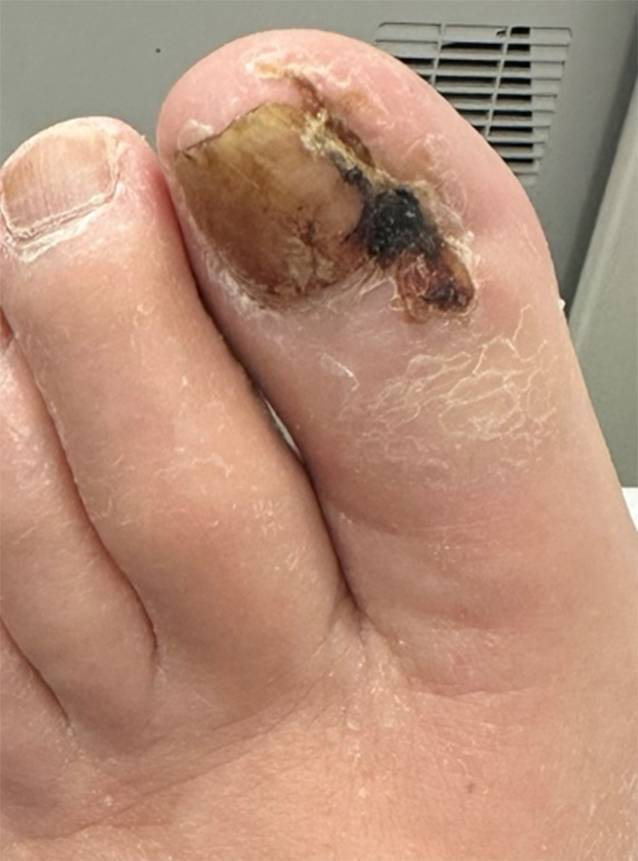
Digitus I des linken Fußes, 38 Tage nach Revaskularisation, Ansicht von ventral

## Hintergrund

Die Prävalenz der peripheren arteriellen Verschlusskrankheit (pAVK) ist altersabhängig. Während die Gesamtprävalenz bei etwa 7–10 % liegt, steigt sie ab dem 70. Lebensjahr auf 15–20 % an, mit einer zunehmenden globalen Tendenz [1]. In dem Kontext kardiovaskulärer Erkrankungen stellt die pAVK eine Manifestation der Atherosklerose oder Atherothrombose in den Extremitäten dar, weshalb ein erhöhtes Kreuzrisiko sowie eine gesteigerte Inzidenz anderer kardiovaskulärer Manifestationen bestehen [1]. Eine 5‑Jahres-Follow-up-Studie der GET-ABI-Studie zeigte eine Verdopplung der Inzidenz von Schlaganfällen (ausgenommen hämorrhagische Schlaganfälle) bei Patienten mit pAVK im Vergleich zu jenen ohne pAVK [2]. Typische kardiovaskuläre Risikofaktoren wie aktiver Tabakkonsum, wurden mit einem erhöhten Risiko für Revaskularisierungsmaßnahmen und für das Auftreten einer „chronic limb threatening ischemia“ (CLTI) in Verbindung gebracht [3]. Nebendiagnosen wie eine koronare Herzkrankheit (KHK), ein vorausgegangener Schlaganfall oder eine begleitende Herzinsuffizienz können das Risiko für das Vorliegen einer pAVK erhöhen und ein entsprechendes Risikoprofil definieren.

Zur adäquaten Diagnostik ist die Durchführung eines Ankle-Brachial-Index (ABI) von entscheidender Bedeutung. Die Sensitivität des ABI zur Erkennung einer pAVK beträgt 90 %, während die Spezifität nahezu 100 % erreicht. Im Vergleich dazu liegt die Sensitivität der alleinigen Palpation des Pulsstatus bei nur 20 %. Die GET-ABI-Studie zeigte, dass ab einem Alter von 65 Jahren jeder 5. Patient einen ABI von < 0,9 oder eine manifeste pAVK aufwies [4]. Das Verhältnis von klinisch asymptomatischen zu symptomatischen Patienten wird mit 2:1 bis 4:1 beschrieben [5].

Angesichts der möglichen Wundheilungsstörungen und einer Verschlechterung der peripheren Perfusion sollte die Durchführung operativer Eingriffe an Beinen und Füßen bei pAVK-Patienten kritisch hinterfragt und in Abwägung der individuellen Befunde infrage gestellt werden [6]. Die Anwendung einer Blutsperre stellt bei vorliegender pAVK eine klare Kontraindikation dar [7]. Für die Erzeugung einer Blutsperre durch pneumatische Kompression wird die Manschette üblicherweise auf 75–100 mm Hg (maximal 350 mm Hg) über den systolischen Blutdruck befüllt [7]. Auch eine Kompressionstherapie mit Strümpfen (20–30 mm Hg) sollte bei einem ABI < 0,5 vermieden werden [8].

Wie im vorliegenden Fallbeispiel gezeigt, führte ein vermeintlich schneller ambulanter operativer Eingriff zu einer akuten Verschlechterung einer pAVK im Stadium IIb, das in ein Stadium IV mit kritischer Extremitätenischämie überging. Im chronischen Stadium der pAVK, bei bereits ausgebauter Kollateralisierung des Gefäßnetzes, bleibt eine akute Symptomatik aus, sodass die Erkrankung häufig unentdeckt bleibt. Daher sollte bei Patienten mit entsprechendem kardiovaskulärem Risikoprofil, die eine Operation der Beine und Füße in Blutsperre oder Blutleere erfordern, stets an das Vorliegen einer pAVK gedacht und bei vorliegender pAVK davon abgesehen werden. Eine Evaluation einer dem Risikoprofil angepassten Anamnese und Diagnostik, wie die Erhebung des Pulsstatus und die Durchführung eines ABI, ist in solchen Fällen richtungsweisend. Sollte der Verdacht auf eine pAVK erhärtet werden, ist eine vorherige gefäßchirurgische Vorstellung empfehlenswert.

## References

[1] Deutsche Gesllschaft für Angiologie, Deutsche Gesellschaft für Gefäßmedizin (2024) S3-Leitlinie zur Diagnostik, Therapie und Nachsorge der Peripheren arteriellen Verschlusskrankheit Bd. 195

[2] Meves SH, Diehm C, Klaus B (2010) Peripheral arterial disease as an independent predictor for excess stroke morbidity and mortality in primary-care patients: 5‑year results of the getABI study. Cerebrovasc Dis. 10.1159/000306640 20375496 10.1159/000306640

[3] Nordanstig J, Behrendt C‑A, Baumgartner I (2024) Editor’s Choice—European Society for Vascular Surgery (ESVS) 2024 Clinical Practice Guidelines on the Management of Asymptomatic Lower Limb Peripheral Arterial Disease and Intermittent Claudication. Eur J Vasc Endovasc Surg 1:9–96. 10.1016/j.ejvs.2023.08.067 10.1016/j.ejvs.2023.08.067 37949800

[4] getABI Study group (2002) getABI: German epidemiological trial on ankle brachial index for elderly patients in family practice to dedect peripheral arterial disease, significant marker for high mortality. Vasa 4:241–248. 10.1024/0301-1526.31.4.241 10.1024/0301-1526.31.4.241 12510548

[5] Debus ES, Walter G‑F (2020) Operative und interventionelle Gefäßmedizin. Springer, Berlin

[6] Tarwnah A, De la Cruz M, Tezval M (2024) Emmert-Plastik. Oper Orthop Traumatol. 10.1007/s00064-024-00843-z 38594591 10.1007/s00064-024-00843-z

[7] Hambloch S, Stegers M, Flender S (2023) Medizintechnische Geräte. In: OTA-Lehrbuch. Springer, Berlin, Heidelberg, S 109–140

[8] Rabe E, Földi E, Gerlach H (2018) Leitlinie: Medizinische Kompressionstherapie der Extremitäten mit Medizinischem Kompressionsstrumpf (MKS), Phlebologischem Kompressionsverband (PKV) und Medizinischen adaptiven Kompressionssystemen (MAK) Bd. 47 (Deutsche Gesellschaft für Phlebologie u. Lymphologie e. V. (DGPL))

